# Protection against Doxorubicin-Induced Cardiac Dysfunction Is Not Maintained Following Prolonged Autophagy Inhibition

**DOI:** 10.3390/ijms21218105

**Published:** 2020-10-30

**Authors:** Ryan N. Montalvo, Vivian Doerr, Oh Sung Kwon, Erin E. Talbert, Jeung-Ki Yoo, Moon-Hyon Hwang, Branden L. Nguyen, Demetra D. Christou, Andreas N. Kavazis, Ashley J. Smuder

**Affiliations:** 1 Department of Applied Physiology and Kinesiology, University of Florida, Gainesville, FL 32611, USA; ryan.montalvo@ufl.edu (R.N.M.); vdoerr@ufl.edu (V.D.); JeungKiYoo@texashealth.org (J.-K.Y.); mhwang@inu.ac.kr (M.-H.H.); branden.nguyen@ufl.edu (B.L.N.); ddchristou@hhp.ufl.edu (D.D.C.); 2 Department of Kinesiology, University of Connecticut, Storrs, CT 06269, USA; ohsung.kwon@uconn.edu; 3 Department of Health and Human Physiology, University of Iowa, Iowa City, IA 52242, USA; erin-talbert@uiowa.edu; 4 School of Kinesiology, Auburn University, Auburn, AL 36849, USA; ank0012@auburn.edu

**Keywords:** adriamycin, anthracycline, chemotherapy, cardiotoxicity, oxidative stress

## Abstract

Doxorubicin (DOX) is a highly effective chemotherapeutic agent used in the treatment of various cancer types. Nevertheless, it is well known that DOX promotes the development of severe cardiovascular complications. Therefore, investigation into the underlying mechanisms that drive DOX-induced cardiotoxicity is necessary to develop therapeutic countermeasures. In this regard, autophagy is a complex catabolic process that is increased in the heart following DOX exposure. However, conflicting evidence exists regarding the role of autophagy dysregulation in the etiology of DOX-induced cardiac dysfunction. This study aimed to clarify the contribution of autophagy to DOX-induced cardiotoxicity by specifically inhibiting autophagosome formation using a dominant negative autophagy gene 5 (ATG5) adeno-associated virus construct (rAAV-dnATG5). Acute (2-day) and delayed (9-day) effects of DOX (20 mg/kg intraperitoneal injection (i.p.)) on the hearts of female Sprague–Dawley rats were assessed. Our data confirm established detrimental effects of DOX on left ventricular function, redox balance and mitochondrial function. Interestingly, targeted inhibition of autophagy in the heart via rAAV-dnATG5 in DOX-treated rats ameliorated the increase in mitochondrial reactive oxygen species emission and the attenuation of cardiac and mitochondrial function, but only at the acute timepoint. Deviation in the effects of autophagy inhibition at the 2- and 9-day timepoints appeared related to differences in ATG5–ATG12 conjugation, as this marker of autophagosome formation was significantly elevated 2 days following DOX exposure but returned to baseline at day 9. DOX exposure may transiently upregulate autophagy signaling in the rat heart; thus, long-term inhibition of autophagy may result in pathological consequences.

## 1. Introduction

Doxorubicin (DOX) is a highly effective chemotherapeutic agent used to reduce tumor burden in a wide variety of cancers [1]. Unfortunately, the clinical use of DOX is restricted due to the development of cardiotoxicity [2,3,4]. Current practices attempt to limit the cumulative dose of DOX in an effort to reduce the incidence of congestive heart failure (CHF) [5]. However, retrospective studies evaluating DOX-related cardiac outcomes indicate that CHF occurs with greater frequency and with lower cumulative doses than previously recognized [5,6]. The onset of cardiomyopathy following DOX chemotherapy negatively impacts long-term cardiac outcomes in cancer survivors and also severely limits treatment options for patients with relapsed or refractory disease [5]. Despite the prevalence and gravity of DOX-induced cardiac dysfunction there are currently no clinically-approved preventative strategies or standard of care practices for the management of DOX-related cardiomyopathy in cancer patients and survivors. Thus, detailed understanding of the molecular mechanisms that drive DOX cardiotoxicity is necessary for the development of cardioprotective therapies.

A dynamic role for autophagy has been proposed in the development of DOX cardiotoxicity [7,8,9]. Although controversy exists regarding autophagy’s complex interaction with pathological processes and its potential to disrupt cellular homeostasis, it is understood that DOX alters the regulation of autophagy within the heart [8]. Under homeostatic conditions autophagy functions to degrade and recycle damaged or senescent organelles, proteins and cellular components. However, continued balance of autophagic signaling is necessary for the sustained health and function of cardiomyocytes [10,11]. Following DOX exposure, the prevailing consensus suggests that autophagy is impaired as the result of an acute increase in autophagosome formation and corresponding suppression of autophagic flux [12].

Although aberrant proteolytic processing by autophagy has been consistently reported in cardiomyocytes exposed to DOX, the physiological consequences of this dysfunction remain unclear [8]. Given the potential for impaired autophagy to promote reactive oxygen species (ROS)-induced cellular damage and mitochondrial dysfunction, [9] targeting autophagy may be an effective strategy to reduce oxidative damage to cardiomyocytes and limit myocardial injury. Thus, this study aimed to clarify the contribution of autophagy to DOX-induced cardiac dysfunction and elucidate the relationship between autophagy and disturbed redox balance by preventing early autophagosome initiation in the heart. Our results uncovered acute benefits to autophagy inhibition that were not observed at a later timepoint.

## 2. Results

### 2.1. Biological Response to dnATG5 and Doxorubicin Exposure

Body weight did not differ initially (initial weight) or four weeks following treatment with autophagy gene 5 (ATG5) recombinant adeno-associated virus (rAAV-dnATG5) or saline (treatment weight) among experimental groups in either the 2- or 9-day experiments (Table 1). Additionally, final weight and heart weight for groups euthanized 2 days following DOX or saline administration were not different. At 9 days, all DOX-treated rats receiving either rAAV-dnATG5 or saline, weighed considerably less and had a significantly reduced heart weight compared to saline-treated rats.

### 2.2. Validation of the Experimental Treatment

**Figure 1:**
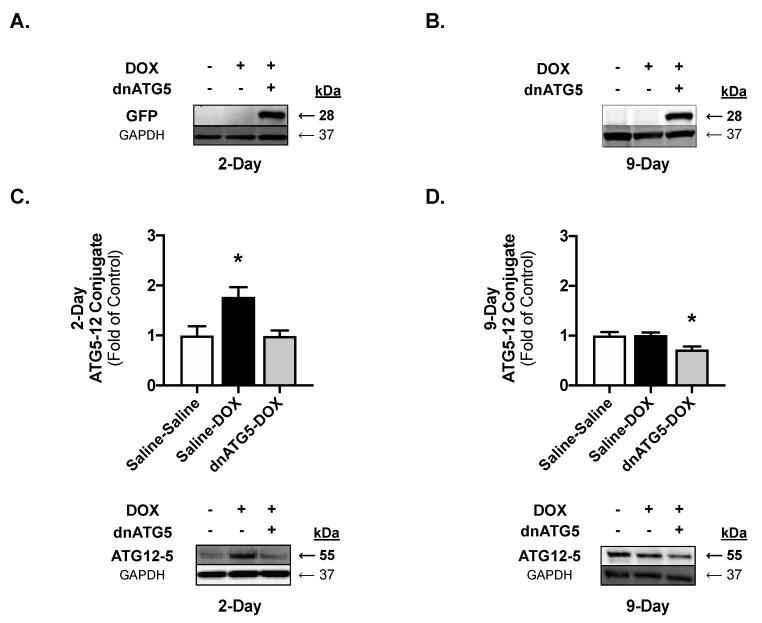
Treatment validation. Western blot to confirm the presence of the rAAV-dnATG5 green fluorescent protein (GFP) tag for the (**A**) 2-day and (**B**) 9-day timepoints. Western blot for the ATG5-ATG12 conjugation product for the (**C**) 2-day and (**D**) 9-day timepoints. Values are presented as means ± SEM, *n* = 7–8/group. Representative Western blot images are displayed below the graphs. * significantly different versus all groups (*p* < 0.05).

Expression of GFP and the ATG5–ATG12 conjugation product in cardiac tissue were determined to confirm the efficacy of our intervention. GFP expression was exclusively detected in the rAAV-dnATG5-treated rats (Figure 1A,B). At the 2-day timepoint, expression of the ATG5-ATG12 conjugate was significantly elevated in the Saline-DOX group compared to Saline-Saline and dnATG5-DOX groups (Figure 1C). At the 9-day timepoint ATG5–ATG12 conjugation was significantly reduced in the dnATG5-DOX rats compared to Saline-Saline and Saline-DOX (Figure 1D).

### 2.3. Inhibition of Autophagosome Formation Protects against Acute DOX-Induced Cardiomyopathy

**Figure 2:**
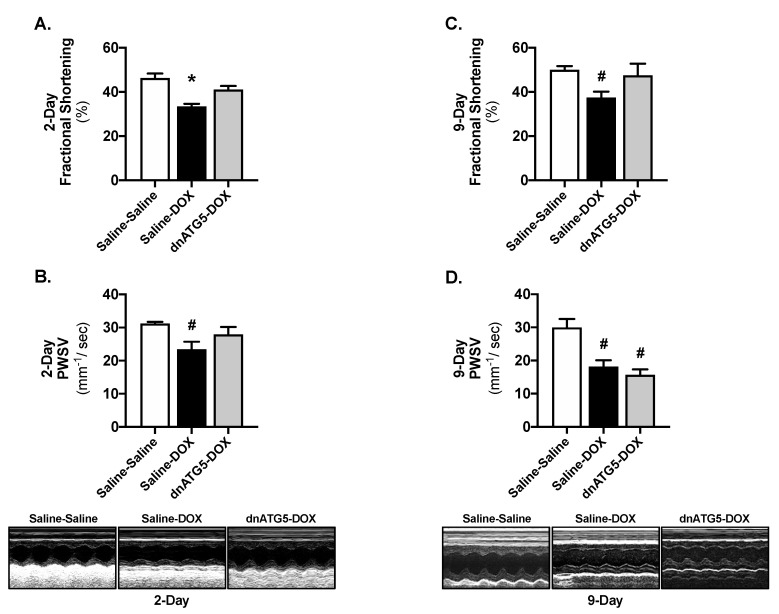
Fractional shortening and posterior wall shortening velocity. Fractional shortening percentage (%) measured at the (**A**) 2-day and (**C**) 9-day timepoints. Posterior wall shortening velocity (PWSV) analyzed at the (**B**) 2-day and (**D**) 9-day timepoints. Representative M-mode images are displayed below the graphs for the 2-day and 9-day timepoints. Values are presented as means ± SEM, n = 6–9/group. * significantly different versus all groups (*p* < 0.05). **^#^** significantly different versus Saline-Saline (*p* < 0.05).

Similarly to previous findings [13,14], our data show that administration of a single injection of DOX (20 mg/kg intraperitoneal injection (i.p.)) results in the rapid development of cardiac dysfunction. Evaluation of left ventricle (LV) systolic function 2 days following DOX revealed a significant reduction in fractional shortening in Saline-DOX rats compared to Saline-Saline and dnATG5-DOX (Figure 2A), and a reduction in posterior wall shortening velocity (PWSV) in Saline-DOX rats compared to Saline-Saline (Figure 2B). Contrastingly, preservation of cardiac function was not retained in the dnATG5-DOX group 9 days post DOX administration. At day 9, fractional shortening was significantly reduced in the Saline-DOX rats compared to Saline-Saline (Figure 2C), and PWSV was diminished in both the Saline-DOX and dnATG5-DOX groups compared to Saline-Saline (Figure 2D).

**Figure 3:**
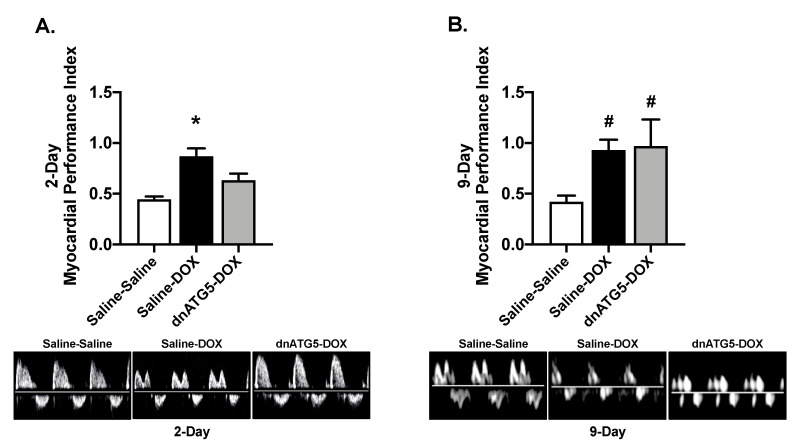
Myocardial performance index. Myocardial performance index was analyzed at the (**A**) 2-day and (**B**) 9-day timepoints. Representative doppler images are displayed below the graph for the 2-day and 9-day timepoints. Values are presented as means ± SEM, n = 5–8/group. * significantly different versus all groups (*p* < 0.05). **^#^** significantly different versus Saline-Saline (*p* < 0.05).

Assessment of LV global diastolic and systolic function revealed a significant increase in myocardial performance index (MPI) in Saline-DOX rats compared to all other groups 2 days post DOX treatment (Figure 3A). However, MPI was significantly increased in the Saline-DOX and dnATG5-DOX groups compared to Saline-Saline at 9 days (Figure 3B). In addition, independent of saline or dnATG5 treatment, DOX resulted in reduced LV septal wall thickness during systole and diastole as well as reduced LV posterior wall thickness during systole compared to Saline-Saline rats at 2 days (Table 2). No differences existed between groups for any parameter of wall thickness at the 9-day timepoint (Table 2).

### 2.4. Inhibition of Autophagosome Formation Protects against Acute DOX-Induced Mitochondrial Dysfunction and ROS Production

**Figure 4:**
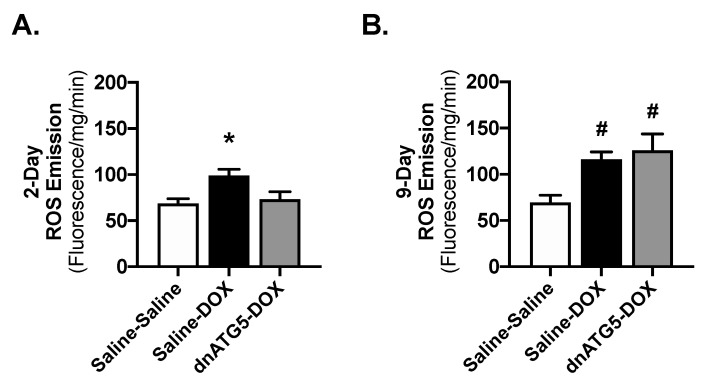
Reactive oxygen species (ROS) emission. H_2_O_2_ emission from permeabilized cardiac muscle fibers at the (**A**) 2-day and (**B**) 9-day timepoints. Values are presented as means ± SEM, *n* = 7–9/group. * significantly different versus all groups (*p* < 0.05). **^#^** significantly different versus Saline-Saline (*p* < 0.05).

Cardiac mitochondrial uncoupling and enhanced rate of ROS generation are directly related to the development of cardiac dysfunction following DOX exposure [13]. Our results confirm these previous findings 2 days following DOX exposure; Saline-DOX rats had a significantly decreased respiratory control ratio (RCR) compared to all other groups, as the result of increased mitochondrial state 4 respiration (Table 3). In addition, ROS production from permeabilized cardiac muscle fiber bundles of Saline-DOX animals produced a significantly greater amount of H_2_O_2_ compared to both other groups (Figure 4A). A total of 9 days following DOX exposure RCR was decreased in both the Saline-DOX and dnATG5-DOX rats compared to Saline-Saline, with no significant differences in state 3 or state 4 respiration among groups (Table 3). H_2_O_2_ emission at 9 days was elevated in the Saline-DOX and dnATG5-DOX groups compared to Saline-Saline (Figure 4B).

## 3. Discussion

DOX is one of the most widely utilized antineoplastic agents that is unfortunately linked to the development of severe cardiac pathology [15,16,17,18,19]. Despite extensive investigation into the molecular mechanisms responsible for DOX cardiotoxicity, a precise understanding remains indeterminate. In particular, autophagy is implicated in the cardiac response to DOX as a result of increased oxidative damage to mitochondria [7,8,9,10,11]. However, conflicting results exist regarding the contribution of autophagy to DOX-induced cardiomyopathy [9]. Therefore, this study was designed to further discern the role of aberrant autophagy signaling following DOX administration by evaluating the acute (2-day) and delayed (9-day) effects of autophagy inhibition on the heart. Our results demonstrate that autophagy signaling is upregulated acutely following DOX administration and that preventing the DOX-induced activation of autophagy resulted in a cardioprotective phenotype. However, sustained inhibition of cardiac autophagy abolishes the beneficial effects of autophagy inhibition due to the transient nature of autophagy signaling upregulation following DOX exposure.

### 3.1. DOX-Induced Autophagy Signaling

Autophagy is a catabolic process responsible for maintaining cell homeostasis through recycling of dysfunctional and long-lived proteins and organelles by lysosomal proteases. Proper regulation of the steps responsible for autophagosome formation, autophagosome–lysosome fusion and autolysosome degradation is required to support controlled degradation of intracellular proteins within this system [20,21]. In the heart, investigation into the role of autophagy during basal conditions has shown that deficiency of lysosome-associated membrane protein 2 (LAMP2), a protein required for formation of the autolysosome, resulted in accumulation of autophagic vacuoles, impaired protein degradation and the development of cardiomyopathy [22,23]. Similar results have been demonstrated with deficient autophagosome formation via ATG5 deletion, indicating an important role for autophagy in the preservation of cardiomyocyte structure and function [24,25]. In contrast, excessive autophagy is also detrimental to cardiac function and is established to promote cardiac dysfunction [26].

While the role of autophagy in DOX-induced cardiac dysfunction remains unclear, the growing consensus is that DOX stimulates the initiation of autophagosome formation in cardiac cells [9]. However, temporal evaluation of autophagy upregulation following DOX administration has begun to reveal fluctuation in the expression of autophagy markers when compared at early and delayed timepoints [12,27,28]. Our results support the hypothesis that upregulation of autophagy markers is transient following DOX administration. Specifically, 2 days following DOX injection we show a significant upregulation of ATG5–ATG12 conjugation in the heart. However, when measured 9 days following injection this marker of autophagosome formation was not elevated as reported at 2 days. These data highlight the importance of time course studies in the development of clinical strategies to prevent DOX cardiac dysfunction, as manipulation of ATG5-ATG12 conjugation using rAAV-dnATG5 was sufficient to prevent the DOX-induced increase at day 2, while the same dose reduced ATG5-ATG12 conjugation below basal levels at day 9.

### 3.2. Cardiac Function and DOX-Induced Autophagy

A direct link between autophagy and DOX cardiotoxicity was first established by Lu et al., when they showed that administration of 3-methyladenine (3-MA), a class III phosphatidylinositol 3-kinase (PI3K) inhibitor, in combination with DOX attenuated cardiac dysfunction [29]. Similar findings using 3-MA to inhibit autophagy have been reported in cultured cardiomyocytes, further supporting the notion that autophagy dysregulation is required for DOX-induced pathology [30,31,32,33,34]. In our study, cardiac function examined following DOX administration in rAAV-dnATG5 treated rats demonstrated similar cardioprotection when compared to pharmacological inhibition of autophagy at the 2-day timepoint. This acute cardioprotection is consistent with the hypothesis that inhibition of autophagosome formation prevents DOX cardiotoxicity by maintaining normal autophagic flux and decreasing demand on the lysosomes [12]. However, we also show cardiac function is not preserved in DOX administered rats when autophagosome formation is knocked down below basal levels in the rat heart. The loss of cardioprotection at the delayed timepoint may be related to temporal changes in autophagy signaling as prevention of pathological ATG5-ATG12 conjugation at 2 days presents as a deficiency in conjugation at 9 days.

### 3.3. Relationship between Autophagy and Oxidative Stress

The accumulation of mitochondrial ROS is involved in the progression of DOX cardiomyopathy via the regulation of UNC-51-like kinase 1 (ULK1) phosphorylation at multiple binding sites [35]. Conversely, cardiac function is preserved when mitochondrial ROS production is attenuated, in part by inhibition of autophagy [36]. Specifically, supplementation with antioxidant compounds in conjunction with DOX therapy has proven effective in the preclinical treatment of DOX cardiotoxicity [13]. ROS production in the cardiomyocytes is proposed to induce autophagy as a means to remove damaged mitochondria [37,38]. However, it is also hypothesized that DOX can directly stimulate autophagy, which in turn can jeopardize the cellular defenses against ROS production [12]. Evidence of this has been shown in skeletal muscle where prevention of DOX-induced autophagy in the soleus was associated with enhanced transcription of antioxidant response element-related genes and increased antioxidant capacity [39]. These beneficial modifications to muscle redox balance resulted in the attenuation of mitochondrial dysfunction and ROS emission [39]. Reduced autophagy initiation in the heart via Beclin 1 haploinsufficiency resulted in a similar attenuation in ROS production, which was associated with a decreased need for autolysosomal protein degradation and improved myocardial performance [12]. Furthermore, transgenic overexpression of Beclin 1 promoted ROS production and exacerbated cardiac dysfunction in DOX treated mice [12]. The relationship between autophagy and ROS accumulation is unclear, but may be related to lysosomal dysfunction and the accumulation of damaged proteins [40,41]. In addition, augmented degradation of functional organelles by accelerated autophagic degradation has also been proposed as the link between autophagy and oxidative stress [42,43]. While further work is necessary to determine the exact interaction between these two processes, our results are consistent with the idea that a regulatory cross-talk exists between autophagy and ROS production. In particular, our data show that preservation of basal autophagy signaling in DOX administered rats prevents mitochondrial oxidative damage, and the reduction of autophagosome formation below basal levels impairs redox balance in the heart.

## 4. Methods

### 4.1. Experimental Animals

Young adult (~6-month-old) female Sprague–Dawley rats were used in these experiments. The current study utilized two experimental endpoints to determine acute (2-day) and delayed (9-day) effects of DOX exposure and autophagy on the heart. Animals in the 2-day and 9-day DOX exposure studies were randomly divided between experimental groups. Autophagy was inhibited via tail vein injection of a recombinant adeno-associated virus expressing a dominant negative mutation of ATG5 (dnATG5) (10^11^ vg). The dnATG5 recombinant adeno-associated virus (rAAV-dnATG5) was created via a K130R mutation that prevents the conjugation of ATG5 to ATG12 [44]. The cytomegalovirus (CMV) promoter and AAV serotype 9 were used to drive gene expression of the rAAV-dnATG5 construct, and the vector was tagged with green fluorescent protein (GFP) to verify its presence in the myocardium. Efficacy of this construct has been previously demonstrated by our group [39,42]. Saline was used as the vehicle and was administered identically to dnATG5. Four weeks following rAAV-dnATG5 or vehicle treatment, DOX (20 mg/kg) or saline (equal volume) were administered as a single intraperitoneal (i.p.) injection. This DOX treatment protocol induces reproducible cardiac dysfunction in female rats, which develops two days following administration [13,14]. All procedures were carried out in compliance with the National Institutes of Health *Guide for the Care and Use of Laboratory Animals* [45], and were approved by the Institutional Animal Care and Use Committees of the University of Florida (IACUC protocol #201207739; 8 January 2013; 2-day DOX exposure study) and University of South Carolina (IACUC protocol #2387-101272-100417; 4 October 2017; 9-day DOX exposure study).

### 4.2. Echocardiography

Transthoracic echocardiography was performed to assess cardiac function (Aplio XV, Toshiba Medical Systems, Tokyo, Japan for 2-day DOX exposure study and LogiQe NextGen, SOUND Technologies, Carlsbad, CA for 9-day DOX exposure study). Under anesthesia with inhaled isoflurane, two-dimensional ultrasound images and M-mode tracings of the left ventricle (LV) were obtained in the parasternal short-axis view at the level of the papillary muscles. Measurements were then performed using techniques as reported previously [13]. In brief, LV fractional shortening and PWSV were used to assess LV systolic function, and the MPI was used as a measurement of combined LV diastolic and systolic function. Measurements of diameters, thicknesses and time intervals were performed in ImageJ (NIH) on 10–15 cardiac cycles and averaged for each rat. Analysis and confirmation of cardiac function was performed by researchers blinded to the experimental groups. Following echocardiography animals were euthanized via overdose of inhaled isoflurane and hearts were excised.

### 4.3. Cardiac Muscle Permeabilization

Permeabilized cardiac muscle fiber bundles were used to measure mitochondrial function and ROS production [13]. Briefly, 5–7 mg sections of LV were placed in a plastic petri dish containing ice cold buffer X (50 mM K-Mes, 35 mM KCl, 7.23 mM K_2_EGTA, 2.77 mM CaK_2_EGTA, 20 mM imidazole, 0.5 mM dithiothreitol (DTT), 20 mM taurine, 5.7 mM ATP, 15 mM PCr and 6.56 mM MgCl_2_, pH 7.1). Muscle fibers were gently but thoroughly separated by a single blinded researcher in ice-cold buffer X to maximize surface area. Permeabilization of the fibers occurred by treatment with 50 µg/mL saponin diluted in buffer X and rotated by full inversion continuously for 30 min at 4 °C. Following permeabilization, fiber bundles were washed for 3 × 5 min in ice-cold buffer Z (105 mM K-Mes, 30 mM KCl, 1 mM EGTA, 10 mM K_2_HPO_4_, 5 mM MgCl_2_-6H_2_O, 0.005 mM glutamate, 0.02 mM malate and 0.5 mg/mL BSA, pH 7.1) by continuous inversion rotation.

### 4.4. Mitochondrial Respiration

Mitochondrial oxygen consumption rate was measured polarographically in water-jacketed respiration chambers maintained at 37 °C (Hanstech Instruments, King’s Lynn, UK) [13]. Following calibration, permeabilized fiber bundles were incubated with 1 mL of buffer Z containing 20 mM phosphocreatine to saturate creatine kinase. Flux through complex I was measured using 2 mM pyruvate and 2 mM malate. The ADP-stimulated respiration (state 3) was initiated by adding 0.25 mM ADP to the respiration chamber. Basal respiration (state 4) was determined in the presence of 10 μg/mL oligomycin to inhibit ATP synthesis. RCR was calculated by dividing state 3 by state 4 respiration.

### 4.5. Mitochondrial ROS Emission

ROS emission in permeabilized muscle fibers was determined using Amplex Red (Molecular Probes, Eugene, OR, USA) [13]. This assay is based on the concept that horseradish peroxidase (HRP) catalyzes the H_2_O_2_-dependent oxidation of non-fluorescent Amplex Red to fluorescent resorufin red. Superoxide dismutase was added to the preparation to convert all superoxide into H_2_O_2_. Although this assay measures all H_2_O_2_ produced in the fiber, previous work has indicated that the predominant amount of ROS production in the permeabilized muscle fiber preparation is released from mitochondria [46,47].

### 4.6. Western Blot Analysis

Cardiac muscle samples were homogenized 1:10 (wt/vol) in 5 mM Tris (pH 7.5) and 5 mM EDTA (pH 8.0) with a protease inhibitor cocktail (Sigma-Aldrich, St. Louis, MO, USA) and centrifuged at 1500 g for 10 min at 4 °C. Supernatant was separated from the pellet and supernatant protein content was assessed using the Bradford method (Sigma-Aldrich). An amount of 20–40 µg of protein were separated using polyacrylamide gel electrophoresis, transferred to nitrocellulose membranes and subsequently incubated with primary antibodies directed against conjugated ATG5-ATG12 (1:500; #4180) and GFP (1:1000; #2956) (Cell Signaling Technologies, Danvers, MA, USA) diluted in Odyssey blocking buffer (LI-COR Biosciences, Lincoln, NE, USA). GAPDH (1:1000; sc47724) (Santa Cruz Biotechnology, Dallas, TX, USA) was used to control for equal protein loading and transfer. Membranes were exposed to Alexa Fluor 680 IgG or 800 IgG (LI-COR Biosciences) secondary antibodies. Imaging and analysis were performed using the Odyssey CLx imaging system and Image Studio software (LI-COR Biosciences).

### 4.7. Statistical Analysis

Results were evaluated by ANOVA with Tukey’s post hoc tests performed to determine differences between the means where appropriate. Significance was established at *p* < 0.05 and all values are reported as means ± SEM.

## 5. Conclusions

DOX accumulation within the myocardium creates a toxic environment that fosters the development of cardiac dysfunction. Although dysregulation of autophagy is an established complication associated with DOX cardiotoxicity, the results from this study offer evidence that manipulation of autophagosome formation does not provide extended benefits as a result of temporal changes in autophagy signaling following DOX administration. Nevertheless, our data do support the accepted view that ROS is a key contributing factor to DOX cardiotoxicity and that a direct interaction between autophagy and oxidative stress exists. Finally, as a result of the transient nature of proteolytic activity following cellular injury, this study emphasizes the need for future work focused on differences in acute versus delayed proteolytic signaling in the development of strategies to combat DOX cardiac dysfunction.

## Figures and Tables

**Table 1:** Body weight and heart weight among experimental groups.

	Saline-Saline	Saline-DOX	dnATG5-DOX
**2-Day**			
Initial weight (g)	278.6 ± 3.3	283.1 ± 5.5	280.1 ± 3.4
Treatment weight (g)	296.5 ± 4.4	302.8 ± 5.1	299.9 ± 4.5
Final weight (g)	295.9 ± 4.5	287.1 ± 6.6	290.5 ± 6.3
Heart weight (mg)	870.5 ± 18.8	792.4 ± 27.3	822.0 ± 28.1
**9-Day**			
Initial weight (g)	268.8 ± 3.6	273.0 ± 5.8	278.3 ± 3.3
Treatment weight (g)	286.5 ± 6.8	287.4 ± 6.0	293.3 ± 4.8
Final weight (g)	282.6 ± 5.6	229.4 ± 13.8 ^#^	229.0 ± 5.9 ^#^
Heart weight (mg)	796.0 ± 30.6	597.5 ± 43.1 ^#^	581.1 ± 13.5 ^#^

Initial weight denotes at time of ATG5 recombinant adeno-associated virus (rAAV-dnATG5) or saline treatment. Treatment weight denotes 4 weeks after rAAV-dnATG5 or saline treatment. Final weight denotes weight at euthanasia. Values are presented as means ± SEM. n = 6–9/group. ^#^ significantly different versus Saline-Saline (*p* < 0.05).

**Table 2:** Differences in cardiac left ventricle septal and posterior wall thickness among experimental groups.

	Saline-Saline	Saline-DOX	dnATG5-DOX
**2-Day**			
SWTd (mm)	1.41 ± 0.03	1.24 ± 0.05 ^#^	1.24 ± 0.04 ^#^
SWTs (mm)	2.54 ± 0.07	2.13 ± 0.08 ^#^	2.25 ± 0.09 ^#^
PWTd (mm)	1.43 ± 0.04	1.36 ± 0.09	1.36 ± 0.05
PWTs (mm)	2.54 ± 0.06	1.90 ± 0.11 ^#^	2.16 ± 0.09 ^#^
**9-Day**			
SWTd (mm)	1.74 ± 0.07	2.08 ± 0.23	1.95 ± 0.17
SWTs (mm)	2.80 ± 0.10	2.85 ± 0.20	2.63 ± 0.18
PWTd (mm)	1.81 ± 0.07	2.04 ± 0.15	2.30 ± 0.27
PWTs (mm)	2.86 ± 0.12	2.91 ± 0.20	3.21 ± 0.36

Septal wall thickness during diastole (SWTd) and systole (SWTs). Posterior wall thickness during diastole (PWTd) and systole (PWTs). Values are presented as means ± SEM. *n* = 6–9/group. ^#^ significantly different versus Saline-Saline (*p* < 0.05).

**Table 3:** Cardiac muscle mitochondria state 3 respiration, state 4 respiration and respiratory control ratio (RCR).

	Saline-Saline	Saline-DOX	dnATG5-DOX
**2-Day**			
State 3 (nmoles O_2_/mg/min)	9.81 ± 0.83	9.75 ± 0.67	10.60 ± 0.66
State 4 (nmoles O_2_/mg/min)	2.13 ± 0.23	3.79 ± 0.24 *	2.67 ± 0.13
RCR (State 3/State 4)	4.93 ± 0.37	2.67 ± 0.26 *	4.06 ± 0.23
**9-Day**			
State 3 (nmoles O_2_/mg/min)	9.50 ± 1.57	8.40 ± 1.12	11.29 ± 1.14
State 4 (nmoles O_2_/mg/min)	2.16 ± 0.41	2.37 ± 0.20	3.21 ± 0.30
RCR (State 3/State 4)	4.53 ± 0.18	3.52 ± 0.27 ^#^	3.54 ± 0.20 ^#^

Values are presented as means ± SEM. n = 6–9/group. * significantly different versus all groups (*p* < 0.05). ^#^ significantly different versus Saline-Saline (*p* < 0.05).
